# Why do pre-clinical medical students learn ultrasound? Exploring learning motivation through ERG theory

**DOI:** 10.1186/s12909-021-02869-4

**Published:** 2021-08-19

**Authors:** Ting-Cheng Wang, Wei-Ting Chen, Yi-No Kang, Che-Wei Lin, Chung-Yi Cheng, Fat-Moon Suk, Hao-Yu Chen, Chin-Wang Hsu, Tsorng-Harn Fong, Wen-Cheng Huang

**Affiliations:** 1 Department of Emergency, School of Medicine, College of Medicine, Taipei Medical University, Taipei, Taiwan; 2 Emergency Department, Department of Emergency and Critical Medicine, Wan Fang Hospital, Taipei Medical University, No.111, Sec. 3, Xinglong Rd, Taipei, 11696 Taiwan; 3 Department of Education and Humanities in Medicine, School of Medicine, College of Medicine, Taipei Medical University, Taipei, Taiwan; 4 Department of Education, Wan Fang Hospital, Taipei Medical University, Taipei, Taiwan; 5 Center for Education in Medical Simulation, Taipei Medical University, Taipei, Taiwan; 6 Department of Internal Medicine, School of Medicine, College of Medicine, Taipei Medical University, Taipei, Taiwan; 7 Department of Internal Medicine, Division of Nephrology, Wan Fang Hospital, Taipei Medical University, Taipei, Taiwan; 8 Department of Internal Medicine, Division of Gastroenterology, Wan Fang Hospital, Taipei Medical University, Taipei, Taiwan; 9 Department of Anatomy and Cell Biology, School of Medicine, College of Medicine, Taipei Medical University, Taipei, Taiwan

**Keywords:** Ultrasound, Undergraduate, ERG theory, Learning motivation

## Abstract

**Background:**

In recent years, point-of-care ultrasound (POCUS) has become an essential field of medical education. Bedside ultrasound has become a necessary skill for clinical physicians. Previous studies have already discussed the importance of advancements in ultrasound education. However, learning motivations for ultrasound education have seldom been analyzed in the literature. For medical students, learning ultrasound could have a relevance for their future career. The Existence, Relatedness and Growth (ERG) theory extended Maslow’s hierarchy of needs through these three concepts. This theory has been widely used in the workplace to analyze employee job performance but has not yet been applied in medical education. In this study ERG theory was applied to analyze pre-clinical medical students’ learning motivation toward ultrasound education.

**Method:**

This mixed method study used online questionnaires consisting of open-ended questions as a data collection tool, and based on these results, both qualitative and quantitative analysis were conducted. Participants answered a series of neutral and open-ended questions regarding their motivations to learn ultrasonography. After data collection, a three-step analysis was conducted based on the grounded theory approach. Finally, the results of the thematic coding were used to complete additional quantitative analysis.

**Results:**

The study involved 140 pre-clinical medical students, and their responses fell into 13 specific categories. The analysis demonstrated that students’ motivations toward ultrasound education were unbalanced across the three ERG domains (*F* = 41.257, *p* < .001). Pairwise comparisons showed that students mentioned existence motivation (MD = 39.3%; *p* < .001) and growth motivation (MD = 40.7%; *p* < .001) more frequently than relatedness motivation. However, there was no significant difference between existence motivation and growth motivation (MD = − 1.4%; *p* = .830).

**Conclusion:**

The results revealed that students placed a high value on existence and growth needs rather than relatedness based on the survey. In addition, the findings suggest that ERG theory can be a useful tool to conduct medical education motivation analysis.

**Supplementary Information:**

The online version contains supplementary material available at 10.1186/s12909-021-02869-4.

## Background

With the emergence of Point-of-care ultrasound (POCUS), ultrasound education has become a significant field of training in both graduate and undergraduate medical education. Ultrasound is a safe and non-invasive diagnostic imaging technology, and the emergence of low-cost, highly portable ultrasonography equipment has extended its use at the bedside. Compared to traditional ultrasound, POCUS emphasizes portability and ease of use. POCUS helps clinicians to make diagnostic decisions based on the interpretation imaging obtained at the bedside. For this reason, it is considered the visual stethoscope of the twenty-first century [1]. POCUS has become a crucial skill for clinical physicians. Previous literature has already discussed the importance of advancements in ultrasound education for medical students [2, 3]. However, these studies mainly focus on learning outcomes obtained through the design of different learning courses [2]. Many other factors that also affect educational outcomes, including learning motivation, but this factor is rarely addressed in the literature.

Motivation is defined as the organized pattern of the goals, beliefs, and emotions that a person is striving for [4]. In general, discussions of motivation rely on needs-hierarchy theory or the Motivated Strategies for Learning Questionnaire (MSLQ) [5, 6]. In the medical education field, Knudsen et al. analyzed medical students’ learning motivation toward learning ultrasound imaging using the Situational Motivation Scale (SMS) [7]. All of the learning motivation analysis approaches mentioned above are applicable to the general population. However, ultrasound is a tool and skill that is closely related to medical students’ future careers. Medical students are very likely to have a specific purpose for learning ultrasound, which stems from considerations related to their future work. Hence, a learning motivation theory was used to target career and job fields. The Existence, Relatedness and Growth (ERG) theory is a motivation analysis framework commonly applied to the study of human motivation in the workplace [8].

Clayton P. Alderfer first proposed ERG theory in 1969 [9]. This theory revised Maslow’s hierarchy of needs into 3 domains: Existence, Relatedness, and Growth. Existence refers to concerns about the basics of life [10]. Relatedness is connected to interpersonal needs for mutual trust and respect. Growth is related to achieving self-value through creativity, productivity, or one’s contributions to the world. ERG theory has been applied to career motivation in many professional fields, yet ERG theory has seldom been used in medical education. In the past, ERG has been widely used in the workplace to analyze employee job performance [8, 11, 12]. This study focused on applying ERG theory to analyze pre-clinical medical students’ learning motivation toward ultrasound education.

## Method

The study was conducted through an elective course to understand and analyze learners’ motivation to learn bedside ultrasonography, and data was gathered through an online open-ended item questionnaire.

### Participants

This mixed method study enrolled third-year undergraduate medical students (medical training in Taiwan consists of a six-year undergraduate curriculum including four preclinical years followed by two clinical years). The participants were all active students at Taipei Medical University and were recruited via email announcement and voluntarily enrolled in this elective course.

### Questionnaire design and development

The process of the questionnaire development is that 6 medical educators described the possible motivation question first and then the 2 consensus meeting was held. The final questions (2 open-ended questions and 5 binary questions) were determined and online questionnaire was made. The questionnaire consisted of a series of non-leading and open-ended questions. (Supplementary Material) Two primary questions (question 4 and 5) were used for the main analysis: “Why do you want to learn ultrasound?” and “What do you expect from the ultrasound course?” Questions 1, 2 and 3 were demographic questions about prior ultrasound learning experience. Questions 6 and 7 were accessory questions for future curriculum design. In addition, the questionnaires were administered completely online to prevent any bias due to the influence of faculty in a face-to-face setting.

### Data analysis

After data collection, an initial qualitative analysis was conducted before the pre-clinical medical students started the ultrasonography course. After the initial data analysis, quantitative results were produced. No specific framework was developed before data analysis to allow the emergence of a theoretical framework from the responses to the questions about learning motivations, thus avoiding bias from the researchers’ subjective tendency and opinion. Three steps for analysis were followed based on grounded theory, an inductive method that provides systematic guidelines for gathering, synthesizing, analyzing and conceptualizing qualitative data for the purpose of theory construction [13]. A feature of this method is the identification of meaningful patterns from qualitative data without any pre-defined framework. This methodology is widely applied in qualitative research to develop an understanding of an unknown phenomenon. In this study, the research team identified relevant elements behind each statement by students at the beginning (open-coding step), and the ERG framework was applied later, during the theme coding phase of the analysis. After the initial open coding, the data was compared and potential theories were identified, and it was determined that the ERG framework could appropriately cover every element found in the open coding step. The final coding was completed with ERG theory. The qualitative coding process utilized is summarized as follows:

The analysis was conducted by a 3-member team: A clinical teacher (emergency physician for 10 years subspecializing in emergency ultrasound and medication education), a medical educator (educational faculty member for 7 years subspecializing in evidence-based medicine and medical education), and a senior learner (third-year emergency medicine resident).

#### First step

Initial simple identification and classification of learning motivations without a specific framework.

#### Second step

The researchers held a consensus meeting for thematic coding based on ERG theory.

#### Third step

The evaluators finished the thematic coding of learning motivations.

Finally, the results of the thematic coding were used to complete the quantitative phase of the analysis. Since research members in this study coded the qualitative statements for quantitative analysis, calculation of inter-coder reliability in content analysis approach was determined as an appropriate method for examining the quality of coding in this study. The content inter-coder reliability in this study was calculated based on the formula by Holsti [14], and extension formula for inter-coder reliability among multiple coders was shown below:

1 $$ \mathrm{Inter}-\mathrm{coder}\ \mathrm{agreement}:\mathrm{pi}=\left(2\times \mathrm{M}\right)/\left(\mathrm{n}1+\mathrm{n}2\right) $$

2 $$ \mathrm{Average}\ \mathrm{mutual}\ \mathrm{agreement}:\mathrm{P}=\sum \mathrm{pi}/\mathrm{N} $$

3 $$ \mathrm{Inter}-\mathrm{coder}\ \mathrm{reliability}:\mathrm{R}=\left(\mathrm{N}\times \mathrm{P}\right)/\left[1+\left(\mathrm{N}-1\right)\mathrm{P}\right] $$

M here refers to number of items that each pair coded similarly, and “n” here refers to total number of items each researcher identified. N here refers to total number of pairs of researchers participating in the content coding.

According to the formula, agreement among pairs ranged from .879 to .958 with average mutual agreement value .910. Finally, content inter-rater reliability among three team members was .968. After completing the thematic coding, quantitative analysis by calculating the descriptive statistics and using the chi-square test, t-test, and multivariate analysis were applied. Descriptive statistics included counts of background information and motivation domains. Chi-square tests were used to analyze differences in the ultrasound learning experience (yes / no) and sex in each motivation domain (yes / no). The t-test was used to explore the differences in ultrasound learning experience (yes / no) and sex in the sum of the motivation categories in each domain. Multivariate analysis was used to compare the sum of the motivation domains and the sum of the motivation categories. For these continuous analyses, the mean difference (MD) was calculated. These analyses were completed in Statistical Product and Service Solutions version 19 for Microsoft Windows. Eta-square was used to show the multivariate analysis’s effect size and set a standard threshold, *p*-value < 0.05, to judge the statistical significance level. The 95% confidence interval to indicate the estimated range was reported.

### Ethics approval and consent

The study was approved by Taipei Medical University Joint Institutional Review Board (TMU-JIRB) for Human Experimentation (IRB TMU-JIRB N201909012).

Since data was collected through a standard post-course survey, the TMU-JIRB suggested that verbal informed consent was sufficient. Informed consent: Informed consent was obtained from all individual participants included in the study.

## Results

The study distributed 140 questionnaires online, and 140 responses were received. Thirty-eight respondents provided answers that were insufficient to conduct a full thematic analysis (such as “interesting” and “want to learn”), but since they met the inclusion criteria, the data from these answers were included in the study for validity analysis.

The research involved 140 pre-clinical medical students, of whom 78 were male, and 62 were female. A total of 19 students had participated in ultrasound learning before the survey. All of them completed the open-ended online study. The qualitative analysis identified 13 categories within three motivational domains (Table 1). The existence domain encompassed four categories. The category “future work requirement” was the most frequently mentioned motivation for learning ultrasound in the existence domain. The relatedness domain contained only two categories. Regarding the growth domain, students’ responses reflected seven categories. No difference was found in sex or ultrasound learning experience in the three domains (Table 2 and Table 3).

**Table 1:** Characteristics and learning experience on each domain

	Ultrasound learning experience	
Item	No ^a^	Yes ^b^
**Sex**		
Male	69	9
Female	52	10
**Existence**		
E1. Future work requirement	68	14
E2. Want to pass current course (non-specific)	2	1
E3. Want to pass current course (knowledge)	13	3
E4. Want to pass current course (skill)	1	0
Number of students mentioning any motivation about existence	76	15
**Relatedness**		
R1. Teacher-student	27	5
R2. Not teacher-student	3	1
Number of students mentioning any motivation about relatedness	30	6
**Growth**		
G1. To be a good doctor in future	10	1
G2. Just want to learn (non-specific)	21	1
G3. Just want to learn (interesting)	17	1
G4. Just want to learn (diverse)	2	2
G5. Just want to learn (improve my current clinical learning)	9	0
G6. Just want to learn (improve my own diagnostic knowledge)	21	3
G7. Just want to learn (improve my own diagnostic skill)	28	5
Number of students mentioning any motivation about growth	84	9

^a^*n* = 121; ^b^ *n* = 19

**Table 2:** Sex and learning experience difference in mean frequency by motivation domain

Domain and					*95%*	*CI*	
population	M	*SD*	*MD*	*t*	*lower*	*upper*	*P*
**Existence by sex**			− 0.198	−1.924	− 0.401	0.006	.056
Male ^a^	.6410	.58051					
Female ^b^	.8387	.63229					
**Existence by learning experience**			0.253	1.694	−0.424	0.549	.093
Novice ^c^	.6942	.60337					
Experienced ^d^	.9474	.62126					
**Relatedness by sex**			0.027	0.365	−0.121	0.175	.716
Male ^a^	.2692	.44643					
Female ^b^	.2419	.43175					
**Relatedness by learning experience**			0.068	0.626	−0.147	0.282	.533
Novice ^c^	.2479	.43361					
Experienced ^d^	.3158	.47757					
**Growth by sex**			−0.099	−0.727	−0.368	0.170	.468
Male ^a^	.8205	.67888					
Female ^b^	.9194	.92857					
**Growth by learning experience**			−0.208	−1.059	−0.597	0.181	.291
Novice ^c^	.8926	.79374					
Experienced ^d^	.6842	.82007					
**Overall by sex**			−0.269	−1.778	−0.569	0.030	.078
Male ^a^	1.7308	.83235					
Female ^b^	2.0000	.95814					
**Overall by learning experience**			0.113	0.508	−0.326	0.551	.613
Novice ^c^	1.8347	.90689					
Experienced ^d^	1.9474	.84811					

a. *n* = 78; b. *n* = 62; c. n = 121; d. *n* = 19. M, mean; MD, mean difference; SD, standard deviation

**Table 3:** Number of students describing specific motivation domains by sex and learning experience

	Sex		Chi-		Ultrasound learning experience		Chi-	
Item	Male ^a^	Female ^b^	square	*p*	No ^c^	Yes ^d^	square	*p*
**Sex**							0.621	.431
Male	–	–	–	–	69	9		
Female	–	–			52	10		
**Existence**			1.742	.187			1.88	.170
No mentioned	31	18			45	4		
Mentioned	47	44			76	15		
**Relatedness**			0.135	.714			0.396	.529
No mentioned	57	47			91	13		
Mentioned	21	15			30	6		
**Growth**			0.004	.947			3.581	.058
No mentioned	26	21			37	10		
Mentioned	52	41			84	9		

^a^ n = 78; ^b^ n = 62; ^c^ n = 121; ^d^ n = 19

### Comparison of the three domains

Overall, 91 (65%), 36 (25.71%), and 93 (66.43%) students mentioned existence motivation, relatedness motivation, and growth motivation for learning ultrasound (Table 3). The test showed that students’ motivations for ultrasound learning were unbalanced (*F* = 41.257, *p* < .001). Pairwise comparisons showed that they mentioned existence motivation (MD = 39.3%; 95% CI, 29.5 to 49.0%; *p* < .001) and growth motivation (MD = 40.7%; 95% CI, 28.5 to 52.9%; *p* < .001) more than relatedness motivation. However, there was no significant difference between existence motivation and growth motivation (MD = − 1.4%; 95% CI, − 14.6 to 11.7%; *p* = .830).(Table 4).

**Table 4:** Pairwise comparison of motivations

		95% CI				Eta-
Comparisons	MD	*Lower*	*Upper*	*F*	*p*	square
**Multivariate tests (overall)**				41.257	< .001	.374
Existence vs Relatedness	0.393***	0.295	0.490			
Existence vs Growth	−0.014	−0.146	0.117			
Relatedness vs Growth	−0.407***	−0.529	−0.285			
**Multivariate tests (existence)**				69.996	< .001	.605
E1 vs E2	.471***	.372	.571			
E1 vs E3	.564***	.476	.652			
E1 vs E4	.579***	.496	.661			
E2 vs E3	.093**	.033	.153			
E2 vs E4	.107***	.055	.159			
E3 vs E4	.014	−.014	.043			
**Multivariate tests (relatedness)**				25.605	< .001	.156
R1 vs R2	.200***	.122	.278			
**Multivariate tests (growth)**				9.540	< .001	.299
G1 vs G2	−.079	−.154	−.003			
G1 vs G3	−.050	−.126	.026			
G1 vs G4	.050	−.004	.104			
G1 vs G5	.014	−.046	.074			
G1 vs G6	−.093	−.170	−.015			
G1 vs G7	−.157	−.238	−.076			
G2 vs G3	.029	−.056	.113			
G2 vs G4	.129	.066	.191			
G2 vs G5	.093	.018	.168			
G2 vs G6	−.014	−.108	.080			
G2 vs G7	−.079	−.181	.024			
G3 vs G4	.100	.039	.161			
G3 vs G5	.064	−.009	.137			
G3 vs G6	−.043	−.130	.044			
G3 vs G7	−.107	−.207	−.008			
G4 vs G5	−.036	−.086	.015			
G4 vs G6	−.143	−.214	−.072			
G4 vs G7	−.207	−.286	−.128			
G5 vs G6	−.107	−.176	−.039			
G5 vs G7	−.171	−.254	−.089			
G6 vs G7	−.064	−.147	.019			

^+^*p* < .10; * *p* < .05; ** *p* < .01; *** *p* < .001; CI, confidence interval; MD, mean difference

### Further comparison of the categories in each motivation domain

In addition, the sub-motivations in each domain were analyzed (Table 4). Regarding the existence domain, E1 was mentioned more frequently than E2, E3, and E4. Moreover, E4 was mentioned less than E2 and E3. Regarding the relatedness domain, R1 was mentioned more frequently than R2.

In the growth domain, the seven sub-motivations, and G7was more frequently mentioned than G1, G2, G3, G4, G5, and G6 .

## Discussion

### Overview

This purpose of this study was to apply ERG theory to analyze pre-clinical medical students’ learning motivation toward ultrasound education.

Most previous studies focused only on the students’ feedback after curriculum implementation to understand the effects of different curriculum designs [15–17]. Most of these studies indicated that motivation increased after curriculum implementation but seldom mentioned students’ motivation before participating in the training. This study is the first to provide a detailed exploration of students’ motivation before engaging with the curriculum to understand why students want to learn ultrasound.

### Learning theories and motivation

There are several learning theories related to adult learning [18]. Focusing on learning motivation, these contemporary theories in medical education include the expectancy-value [19], attribution [20], social-cognitive [21], goal orientation [4, 22], and self-determination theories [23]. These previously described findings seem unsuitable for the analysis of qualitative data from learners. Unlike traditional theories, ERG theory can be applied to extract more qualitative data from learners’ responses.

### Key findings

The study suggests that existence needs and growth needs were more significant than relatedness needs concerning students’ motivation to learn ultrasound. The findings indicate that students learn ultrasonography voluntarily because they consider ultrasound skills to be closely connected to their future careers as doctors. In the existence needs subgroup analysis, most students mentioned the words “future” and “clinical”. In particular, the categories (E1) Future work requirement and (G7) Just want to learn (improve diagnostic skills) dominated, even though ultrasound training is not required for medical undergraduates in Taiwan.

### Participant learning motivation

None of the students in the study had started their clinical training, and had little previous ultrasound experience. However, they showed great interest in learning because they anticipated future applications of ultrasound. This interest suggests that students are already aware of the increasing importance of bedside ultrasonography, which has become a trend in recent years. Concerning growth needs, learning ultrasonography skills has great importance for students. This phenomenon may be related to the fact that unlike X-ray and computed tomography (CT) examinations, ultrasound is an operator-dependent diagnostic tool. Ultrasonographic skills development is of great importance in ultrasound education and is also affected by learning motivation. Previous studies on ultrasound education mainly focus on performance [2, 15–17]. Unfortunately, the learning motivations for learning ultrasound were not well established.

### Motivation analysis

In performing further motivation analysis, some results were unexpected. The first finding was that relatedness needs were less salient than existence needs and growth needs. Relatedness needs were expected be as important as the other types of needs, particularly in the Taiwanese cultural context in which Confucianism has a far-reaching influence. Interpersonal relationships are vital and affect learning motivation in individuals of Chinese background [24]. Surprisingly, the research did not reflect this, possibly because of students’ awareness that ultrasound skills were essential for their future work. The researchers concluded this was not due to a reduction in the relatedness needs in Chinese culture but that the widespread nature of ultrasound applications has promoted increased existence and growth needs. Students’ learning motivation is thus less affected by interpersonal factors.

### Sex and motivation

The second finding was that relatedness needs did not differ between the sexes. It was initially assumed that sex would influence the motivation of medical students to learn ultrasound. Previous studies have suggested that traditional sex role stereotypes dictate that men seem more inclined toward mathematics, science and rationality. In contrast, women have a more substantial interest in language arts and writing [25]. Thus, sex may also affect learning motivation. However, the research revealed that there were no sex differences concerning the relatedness motivation to learn sonoanatomy. This result may be explained by Bandura’s Triadic Theory of Learning. Human learning is affected by environmental factors, personal factors, and behavioral factors rather than by a single intrinsic tendency and is influenced by a person’s surroundings [26]. Therefore, sex is not sufficient to influence the motivation of medical students to learn ultrasound. The results strengthen the environmental explanation (the increasing trend in ultrasound applications in clinical practice) mentioned above. Ultrasound is a crucial skill in students’ future careers.

### ERG theory in ultrasonography learning

Because of the use of different training models and differences in medical undergraduates’ needs and perspectives, ERG theory was employed to explore pre-clinical medical students’ motivation to learn clinical skills. The analysis revealed that pre-clinical medical students are more focused on existence and growth needs. Thus, meeting these needs when developing a new course or curriculum should be emphasized. In addition, it is necessary to motivate students and offer future-oriented learning that is effective and efficient. This qualitative analytic study following grounded theory methodology confirms that ERG theory can be applied to explore learning motivation for professional development. The ERG theory may also be adapted to analyze learners’ motivation in other healthcare professions.

### Limitations

First, the online questionnaire’s non-directed, open-ended nature led to some replies being ambiguous, and their true meaning could not be ascertained. Thus, the interpretation may not have necessarily represented the respondents’ actual thoughts. Second, the study was conducted in single institution and in a culturally homogeneous group. The diversity of the sample was insufficient to make more profound generalizations to other cultural contexts. Medical students’ motivation to learn sonoanatomy may differ by school, region, or culture. Third, this research did not consider individual medical students’ background differences and their potential impact on learning motivation.

## Conclusion

This study revealed several meaningful findings. First, students placed a very high value on existence needs and growth needs but not on relatedness needs. Second, in our subgroup analysis, no sex difference was noted with respect to relatedness. Based on this survey of learning motivation for ultrasonography skills, applying ERG theory in the context of medical student and medical personnel education to analyze students’ skill-learning motivation could lead to a better understanding. of learners’ interest in developing their skills.

## Supplementary Information

**Additional file 1.**

## Data Availability

The datasets used and/or analyzed during the current study are available from the corresponding author on reasonable request.

## Abbreviations

POCUS: Point of Care Ultrasound
ERG: Existence, Relatedness, Growth
MSLQ: Motivated Strategies for Learning Questionnaire
SMS: Situational Motivation Scale
MD: Mean Difference
CT: Computed Tomography
