# Supplementary material for: Why do pre-clinical medical students learn ultrasound? Exploring learning motivation through ERG theory
Source: BMC Med Educ. 2021 Aug 19;21:438. doi: 10.1186/s12909-021-02869-4 (PMC8375120; doi:10.1186/s12909-021-02869-4)
Supplement: Supplementary file 1 — Additional file 1. [file 12909_2021_2869_MOESM1_ESM.docx]

Supplementary Material:

**Questionnaire questions before Sonoanatomy course**

| **1.** | Is it your first time to take an ultrasound course? |
| --- | --- |
| **2.** | Did you know that there are ultrasonic anatomy courses abroad? |
| **3.** | Have you attended a workshop or course related to ultrasound? |
| **4.** | Why do you want to learn ultrasound? |
| **5.** | What do you expect from this course? |
| **6.** | Which part of the ultrasound anatomy are you most interested in? |
| **7.** | Are you worried that the integration of ultrasound into anatomy courses will increase the burden of learning? |
